# Case of a Wound in the Perineum, Produced by the Point of a Plough-Share

**Published:** 1820-11

**Authors:** M. Lesage

**Affiliations:** Argentan.


FOR THE LONDON MEDICAL AND PHYSICAL JOURNAL.

*Case of a Wound in the Perineum, produced by the Point of a Ploughshare.* By M. LESAGE, of Argentan. Communicated to the "Société de l'Ecole de Médecine de Paris."

A BOY, twelve years of age, was left to hold, in the absence of the ploughman, two young and vigorous horses, which were attached, laterally, to a plough. The horses, being frightened by some object, took flight at full gallop, and threw down the boy between their feet and the wheels of the plough: he fell on his left side, with his thighs crossed and bent on the trunk. In this position he was struck in the perineum by the point of the plough-share, which had been newly sharpened, and which produced a deep laceration in the part just mentioned. The hemorrhage was moderate, and the boy was able to walk to his home, which was not far distant. Four days after the accident I was called to see him. I found a laceration in the perineum, on the left of the raphe, which extended from the tuberosity of the ischium to the arch of the pubes. The edges of the wound were smooth, hard, and tumefied: the sphincter of the anus was not injured. Part of the feces, and almost the whole of the urine, passed out through the wound; only a few drops of the latter came through the urethra when he made great efforts to expel it. The hypogastric region was tense and painful. He had but little fever.

The symptoms left no reason to doubt that the rectum and the urethra had been torn; but the degree of irritation present at this time did not permit me to resort to the means necessary in order to ascertain the extent of the lesion of these parts.

The patient had been bled in the first instance. The consequent accidents were combatted by a severe abstinence from food, a laxative drink, emollient glysters, hip-baths, fomentations of the abdomen, and poultices to the perineum. In six days the symptoms began to be alleviated.

The use of the catheter was indispensable, but this was attended with great difficulties: the instrument had hardly passed beyond the arch of the pubes, when it became engaged in a laceration of the urethra, and thence passed into the rectum, the anterior paries of which was torn to an extent of eighteen lines. This false passage could be avoided only by giving a great degree of curvature to the instrument, and by pressing the point of it towards the arch of the pubes and upper paries of the canal in the passing of it. The elastic gum catheter was defective for this purpose, because of its great flexibility; and, although it were fixed by all the means in use, it escaped from the bladder whenever the patient voided his feces, and even when he turned in bed in his sleep. It then passed into the rectum, whence its point, being bent by the weight of the fecal matters, came out at the anus. After this accident had recurred several times, this instrument was replaced by a silver catheter, having the form of the italic *S*. Tents of lint, covered by simple digestive ointment, were passed into the rectum by the anus. The wound in the perineum was dressed superficially.

As soon as the urine was constantly passed through the catheter, healthy suppuration became established, and the severe symptoms shortly disappeared. A few drops of urine continued for some time to flow from the wound, having passed between the urethra and the catheter, which produced some degree of hardness in the edges of the laceration. This inconvenience was obviated by leaving the catheter always unstopped. By these measures, and others calculated to remove various accidents as they arose, the wound in the perineum perfectly cicatrized in seventy-two days. The use of the catheter was continued for a month after this time; since when the boy has passed his urine by the natural passage, and seems to be in every respect perfectly well.

